# International Cardiovascular Research Journal and Its Contribution to Iran’s Scientific Growth

**DOI:** 10.17795/icrj-20449

**Published:** 2014-09-01

**Authors:** Mohammad Javad Zibaeenezhad, Mahyar Ahmadpour-B

**Affiliations:** 1 Cardiovascular Research Center, Shiraz University of Medical Sciences, Shiraz, IR Iran; 2 Cardiovascular Engineering Laboratory, Biomedical Engineering Department, Amirkabir University of Technology, Tehran, IR Iran

**Keywords:** Cardiovascular, PubMed

International Cardiovascular Research Journal has been recently indexed in PubMed. In order to evaluate the role of this journal in global and international scientific growth, the scientific growth of cardiovascular researches in the past 13 years has been evaluated. In this way, one can compare the role of this journal in Iran and Iran’s role in the global scientific growth rate.

**Figure 1.**
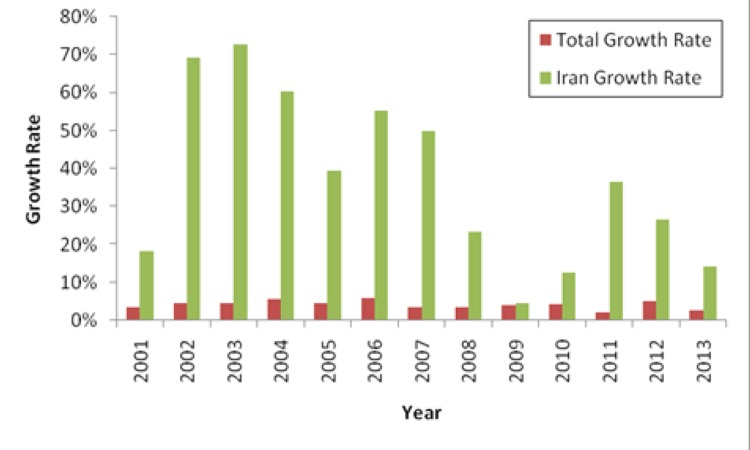
The Annual Scientific Growth Rate in Cardiovascular Researches in Iran and the World

Each year, new medical inventions and discoveries all around the world have major influences on increase of public health and decrease of treatment expenditures. It is obvious that in case the results of a study have not been published, that study has not probably been done or reached any result. Therefore, in evaluation of scientific researches, one may think that only published (and indexed) articles should be taken into account. The number of officially indexed articles in acceptable databases is considered as a manifestation of progression in any field. In the field of medical sciences, PubMed is one of the main databases in which each paper is valuable. In addition, the number of indexed papers in PubMed is considered as a good criterion for determining the progresses in medical sciences. This study aims to identify the scientific outcomes in the field of cardiovascular researches both in Iran and around the world during the past 13 years. According to the statistics, more than 32000 articles were indexed in PubMed during 2000. This figure increased to more than 54000 in 2013, showing a 69% increase during 13 years. Moreover, 11 articles from or about Iran were indexed in this field during 2000, while this measure was reported to be 550 in 2013, revealing a 5000% increase in this area. A year-to-year comparison indicates that Iran’s publications growth rate in this field has always been higher than that of the world. This implies that cardiovascular sciences have a higher rate in Iran compared to the world. Figure 1 depicts the results of an annual comparison between Iran and the world. In addition, Table 1 shows the exact values of the comparisons presented in Figure 1.

**Table 1.** The Annual Number of Articles in the Field of Cardiovascular Researches (1)

Publication Year	Total Articles in PubMed	Articles from or about Iran in PubMed
2000	32494	11
2001	33610	13
2002	35117	22
2003	36766	38
2004	38811	61
2005	40554	85
2006	42950	132
2007	44433	198
2008	46042	244
2009	47841	255
2010	49876	287
2011	50911	392
2012	53474	496
2013	54903	566

International Cardiovascular Research Journal is a quarterly journal indexed in PubMed which publishes about 40 articles every year. Considering this journal’s broad range of authors, it has a significant role in increasing Iran’s scientific share and can play even a more significant role in this regard in future.
